# Latent profile analysis of rehabilitation motivation in Chinese patients with stroke: a cross-sectional study

**DOI:** 10.3389/fpsyg.2025.1614528

**Published:** 2025-08-26

**Authors:** Rong Tang, Qiuxue Sun, Juan Li, Xiaowen Jiang, Shuxian Liu, Xi Chen, Yumei Lv

**Affiliations:** ^1^Department of Nursing, Harbin Medical University, Harbin, China; ^2^Department of Nursing, Affiliated Hospital of Changchun University of Traditional Chinese Medicine, Changchun, China

**Keywords:** stroke, rehabilitation motivation, latent profile analysis, influencing factors, category characteristics

## Abstract

**Objective:**

This study used latent profile analysis to identify distinct profiles of rehabilitation motivation among Chinese patients with stroke and explored the multidimensional predictors of rehabilitation motivation across different patient subgroups based on the biopsychosocial medical model.

**Methods:**

From September 2024 to January 2025, 328 patients with stroke were recruited from the rehabilitation departments of three tertiary hospitals in China using convenience sampling. Data collection included (1) a general information questionnaire, (2) Chinese version of the Stroke Rehabilitation Motivation Scale, (3) Modified Barthel Index, (4) the National Institutes of Health Stroke Scale, and (5) Kessler Psychological Distress Scale. Data were analyzed using Mplus version 8.3 and SPSS version 27.0.

**Results:**

Three latent classes of rehabilitation motivation were identified among patients with stroke: (1) Low Rehabilitation Motivation-Intrinsic Drive Deficiency (Class 1, 30.2%), (2) Moderate Rehabilitation Motivation-Extrinsic Drive Stability (Class 2, 39.0%), and (3) High Rehabilitation Motivation-Intrinsic Drive Sufficiency (Class 3, 30.8%). Multiple logistic regression indicated that age, monthly household income, ADL, severity of neurological impairment, and psychological distress were significant predictors of different rehabilitation motivation classes.

**Conclusion:**

This study identified significant heterogeneity in the rehabilitation motivation profiles of patients with stroke. Healthcare professionals should implement targeted interventions based on the distinct motivational profiles of patients with stroke during their rehabilitation process, with the aim of effectively mobilizing their intrinsic motivation to participate in rehabilitation therapy.

## 1 Introduction

Cerebral stroke is the second leading cause of mortality and third leading cause of disability worldwide, posing a particularly severe threat to the health of Chinese adults. It has long remained the top contributor to death and disability in China's disease spectrum (GBD 2019 Stroke Collaborators, 2021; Hu et al., 2020). While advancements in diagnostic and therapeutic technologies have significantly reduced acute-phase mortality rates, up to 70% of stroke survivors still experience varying degrees of functional impairment, resulting in severe compromises in their ADL and placing substantial caregiving burdens on families and society (Meadmore et al., 2019). As a typical chronic disabling condition, stroke rehabilitation is characterized by a prolonged duration and multifaceted needs. Evidence-based medicine demonstrates that early standardized rehabilitation training can effectively improve motor functions, alleviate psychological complications, and facilitate the restoration of social participation capabilities (Li et al., 2024). However, in clinical practice, the intensity and duration of rehabilitation training for most patients with stroke fail to meet the levels required for significant functional improvements (DiPasquale et al., 2022). Compared with age-matched healthy individuals, adherence of patients with stroke to sustained exercise remains suboptimal (Yumei et al., 2023; Smith et al., 2024), mainly due to insufficient rehabilitation motivation.

Geelen and Soons (1996) were the first to introduce motivation into rehabilitation research, emphasizing that implementing rehabilitation goals depends on patients' motivation levels to participate in therapeutic interventions. Previous studies have shown that rehabilitation motivation not only plays a critical role in enhancing adherence to rehabilitation regimens, improving motor functions, delaying post-stroke frailty, and promoting cognitive and emotional recovery among patients with stroke (Ishida et al., 2023; Gangwani et al., 2022) but also serves as a key determinant of independence in ADL (Cheong et al., 2020). These findings highlight the pivotal role of rehabilitation motivation throughout the recovery process. However, due to neuroplastic alterations in the brain, active engagement of patients with stroke in rehabilitation training remains suboptimal. Research indicates that disease-related psychological stress often leads to diminished motivation in this population, resulting in a “motivation-behavior” disconnect phenomenon (Feng et al., 2022). Rehabilitation data revealed that while ~65% of patients with stroke receive inpatient rehabilitation, over half exhibit insufficient autonomy and participation during the process (Winstein et al., 2016). Notably, community-dwelling patients with stroke experience decreased rehabilitation motivation compared with their inpatient status (Zaw et al., 2022) with 56.7% failing to maintain rehabilitation exercises after discharge (McCracken, 2013). These observations highlight the prevailing passive treatment model in current rehabilitation practices, in which patients' subjective initiatives remain underutilized. This contrasts sharply with modern neurorehabilitation principles that emphasize “proactive rehabilitation” as the optimal paradigm.

Motivation for rehabilitation has garnered significant attention both domestically and internationally. However, the motivation levels of patients with stroke remain suboptimal, and the existence of inter-individual variability in rehabilitation motivation warrants further exploration. Rapoliene et al.'s (2018) study indicated that rehabilitation motivation in the early post-stroke phase diminishes as the disease progresses. The findings of Oh et al.'s (2020) study, using a self-developed Rehabilitation Motivation Questionnaire, revealed moderate motivation levels among middle-aged patients with stroke. Similarly, Tan et al. (2023) employed the unidimensional Motivational Orientation for Rehabilitation Engagement (MORE) scale and reported moderate motivation levels in elderly patients with stroke. These studies collectively highlight a predominant trend in prior research to conceptualize rehabilitation motivation as a unitary construct, categorizing it simply as “high, medium, or low” based on total scale scores, while overlooking its latent heterogeneous characteristics. Latent Profile Analysis (LPA) is a person-centered statistical methodology (Blanken et al., 2020). Fundamentally, it operates as a classifier that assigns individuals to distinct subgroups based on patterns in their responses to a set of observable indicators (e.g., attitudinal measures, behavioral data, or other quantitative traits). By employing LPA, researchers can elucidate meaningful differences across latent population segments, enabling the development of tailored interventions and support strategies for each identified group. This method has demonstrated unique advantages in stratified analyses within mental health and behavioral medicine; however, its application in studying rehabilitation motivation among patients with stroke remains unreported.

Engel's biopsychosocial model elucidates the multidimensional interactive mechanisms underlying disease development, emphasizing the synergistic roles of biological, psychological, and social factors across all disease stages, including prevention, onset, progression, treatment, and rehabilitation (Engel, 1980). This theoretical framework provides critical insights into the pathological mechanisms of chronic conditions, as these illnesses often interact in a complex manner with patients' lifestyles, psychosocial stressors, and psychological resilience. As a prototypical chronic disabling condition, the rehabilitation trajectory of patients with stroke is embedded within this multidimensional system. Rehabilitation motivation, the core driver of functional recovery, is inevitably subject to dynamic modulation by physiological functions, psychological fluctuations, and social support networks. Therefore, adopting a biopsychosocial integrative perspective to dissect the characteristics of rehabilitation motivation across different stroke patient subgroups could lay a theoretical foundation for the rapid identification of distinct motivational profiles and implementation of targeted interventions.

In summary, existing research has predominantly focused on the overall levels of rehabilitation motivation while neglecting the population heterogeneity of this construct among patients with stroke. Consequently, this study aimed to (1) apply LPA to classify patients with stroke into subgroups based on distinct rehabilitation motivation profiles and (2) explore the multidimensional predictors of rehabilitation motivation across these subgroups within the biopsychosocial framework. These objectives will inform clinical practitioners in designing tailored rehabilitation interventions and improving long-term functional outcomes for patients with stroke.

## 2 Methods

### 2.1 Study design, participants, recruitment

This study was a cross-sectional study. From September 2024 to January 2025, patients with stroke were recruited from the rehabilitation departments of three tertiary hospitals in Heilongjiang Province. The inclusion criteria were as follows: (1) meeting the diagnostic criteria for stroke outlined in the Diagnostic Guidelines for Major Cerebrovascular Diseases in China (2019) (Chinese Medical Association, Chinese Stroke Society, 2019), confirmed by CT or MRI with corresponding reports provided; (2) aged 18 years or older; (3) admitted to the rehabilitation department between 2 weeks and 6 months after the onset of non-acute stroke; and (4) providing informed consent and voluntarily participating in the study. The exclusion criteria were as follows: (1) severe dysfunction of the heart, liver, kidneys, or other organs or having malignant tumors; and (2) cognitive or mental impairments that prevent cooperation.

This study was approved by the Ethics Committee of Harbin Medical University (HMUDQ20231116221). To ensure voluntary participation and data integrity during the data collection phase, the participants were provided with a comprehensive informed consent form that clearly outlined the study's purpose, procedures, potential risks, and benefits, while emphasizing the voluntary nature of their participation.

### 2.2 Sample size

The primary statistical method employed in this study was LPA. Previous research has indicated that LPA requires a minimum sample size of 300–500 participants (Ferguson et al., 2019). Accordingly, 340 patients with stroke were recruited, which aligned with the sample requirements of this study.

### 2.3 Method of data collection

#### 2.3.1 Measurement of rehabilitation motivation

The Chinese version of the Stroke Rehabilitation Motivation Scale (SRMS) was used to assess the rehabilitation motivation levels of patients with stroke. The scale was originally developed by White et al. (2012) and later adapted into Chinese by Zhao et al. (2023) from the Nursing School of Qingdao University. Authorization for using the Chinese version was obtained from the original adaptors, and a formal scale authorization agreement was signed. This scale comprises seven dimensions with 28 items, including three negatively worded items (Items 5, 12, and 23). After reversing and recoding the negatively worded items, the total score ranged from 28 to 140, with higher scores indicating greater rehabilitation motivation in patients with stroke. In this study, the scale demonstrated good reliability and validity, with a Cronbach's α coefficient of 0.906.

#### 2.3.2 Measurement of activities of daily living

Activities of daily living (ADL) were assessed using the Modified Barthel Index (MBI). This scale was developed by Shah et al. (1989) to assess ADL in patients with stroke. It consists of 10 dimensions with a maximum score of 100, with higher scores indicating better functional abilities in daily life. In this study, the scale demonstrated good reliability and validity, with a Cronbach's α coefficient of 0.895.

#### 2.3.3 Measurement of the severity of neurological impairment

National Institutes of Health Stroke Scale (NIHSS) was used to assess the severity of neurological impairment in patients with stroke. This scale includes 11 items, such as level of consciousness, gaze, visual fields, and facial muscle function. The score ranges from 0 to 42, with higher scores indicating more severe neurological impairment. A total score of <5 indicates mild neurological impairment, whereas a score of 5 or higher indicates moderate-to-severe neurological impairment. Owing to its high internal consistency, good inter-rater reliability, test-retest reliability, and construct validity, it is widely used to evaluate the degree of neurological impairment in patients with clinical stroke (Brott et al., 1989).

#### 2.3.4 Measurement of psychological distress

The Kessler Psychological Distress Scale (K10) was used to assess the psychological distress in patients with stroke. This scale was originally developed by Kessler et al. (2002) and later adapted into Chinese by Zhou et al. (2009), who also conducted reliability and validity tests. It consists of 10 items with a total score ranging from 10 to 50, with higher scores indicating more severe psychological distress. In this study, the scale demonstrated good reliability and validity, with a Cronbach's alpha coefficient of 0.800.

#### 2.3.5 Measurement of other variables

Based on a review of the literature and expert opinions of the research team, we screened and proposed including the following biological, psychological, and sociological factors based on the biopsychosocial medical model: age, gender, type of stroke, disease duration, number of strokes, ADL score, NIHSS score (biological factors), psychological distress (psychological factor), monthly household income, education level, marital status, and medical expense payment method (sociological factors).

### 2.4 Statistical analysis

SPSS 27.0 was used for data statistical analysis, and Mplus 8.3 software was used for LPA. The statistical inference alpha value was set at a significance level of 0.05, and the *P*-value was two-tailed. The chi-square test was used to compare the differences in biopsychosocial factors among different categories of patients with stroke. Multivariate logistic regression analysis was used to determine predictors of potential categories of rehabilitation motivation in patients with stroke. The optimal number of profiles was selected based on the following model fit indices: Akaike Information Criterion (AIC), Bayesian Information Criterion (BIC), sample-adjusted Bayesian Information Criterion (aBIC), Lo-Mendell-Rubin Test (LMRT), bootstrapped likelihood ratio test (BLRT), and entropy. The smaller the AIC, BIC, and aBIC values, the better the model. If the *P*-values of both LMRT and BLRT reach a significant level, this indicates that the k-profile model is better than the k-1 profile model. Entropy ranges from 0 to 1 and is generally required to be >0.80. The closer it is to 1, the higher the classification accuracy.

## 3 Results

### 3.1 LPA of rehabilitation motivation among patients with stroke

As shown in Table 1, using the scores for the seven dimensions of rehabilitation motivation as explicit indicators, the number of categories increased to five. AIC, BIC, and ABIC decreased as the number of categories increased, and the entropy values were all >0.900. When the number of categories was five, LMRT did not reach a significant level, indicating that Model 4 was better than Model 5. When the number of categories was four, the entropy value decreased from 0.971 to 0.946. Given that “the closer the entropy value is to 1, the more accurate the classification,” and combined with clinical practice, it was inferred that Model 3 was the optimal model.

**Table 1:** Fit indices of LPA for stroke patients' rehabilitation motivation profiles (*N* = 328).

**Profiles**	**AIC**	**BIC**	**ABIC**	**Entropy**	**LMRT**	**BLRT**	**Proportion**
1	12087.186	12140.289	12095.881				1.0
2	10281.489	10364.936	10295.152	0.916	0.4063	0.000	0.481/0.518
**3**	**8972.489**	**9086.28**	**8991.12**	**0.971**	**0.0000**	**0.000**	**0.302/0.308/0.390**
4	8774.092	8918.227	8797.692	0.946	0.0067	0.000	0.286/0.180/0.256/0.277
5	8692.844	8867.323	8721.412	0.934	0.3711	0.000	0.247/0.070/0.256/0.149/0.277

The bold values indicate the optimal model fit indices.

**Figure 1:**
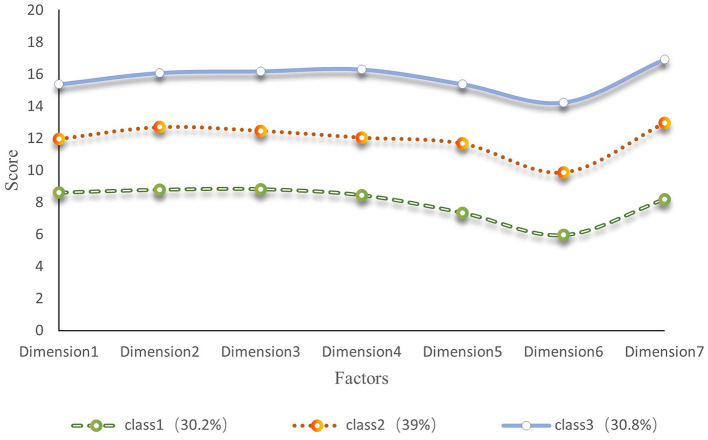
Latent profile characteristics of rehabilitation motivation in stroke patients (seven dimensions of rehabilitation motivation x-axis labels: a motivation, external regulation, introjected regulation, identified regulation, intrinsic motivation-cognition, intrinsic motivation-stimulation, intrinsic motivation-accomplishment, *N* = 328).

**Figure 2:**
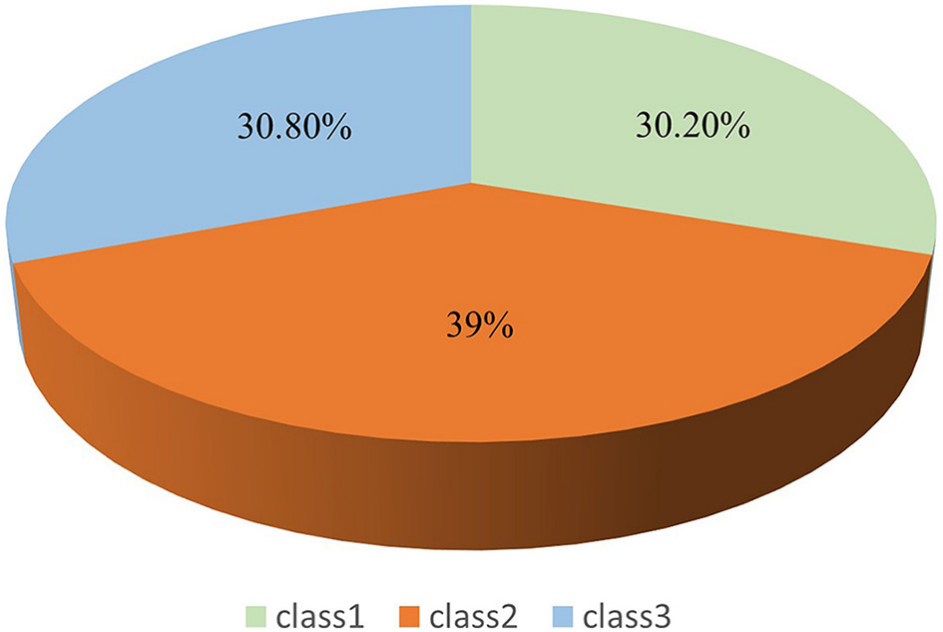
The distribution of three rehabilitation motivation classes.

Figure 1 shows that the first category has the lowest overall mean score for rehabilitation motivation and the lowest mean scores for the dimensions of intrinsic motivation-cognition, intrinsic motivation-stimulation, and intrinsic motivation-achievement. Based on the explanation of the motivational mechanism by Self-Determination Theory (Ryan and Deci, 2017), it was named “Low Rehabilitation Motivation-Intrinsic Drive Deficiency (Class 1, 30.2%).” The second category had a moderate overall mean score for rehabilitation motivation, with small differences in the mean scores for the dimensions of extrinsic motivation-introjection, extrinsic motivation-regulation, and extrinsic motivation-identification, and was named “Moderate Rehabilitation Motivation- Extrinsic Drive Stability (Class 2, 39%).” The third category had the highest overall mean score for rehabilitation motivation and the highest mean scores for the dimensions of intrinsic motivation-cognition, intrinsic motivation-stimulation, and intrinsic motivation-achievement and was named “High Rehabilitation Motivation-Intrinsic Drive Sufficiency (Class 3, 30.8%).” An LPA graph was drawn based on the classification results. Figure 2 clearly illustrates the distribution of three rehabilitation motivation classes.

### 3.2 Disparity analysis of latent profiles of rehabilitation motivation in patients with stroke

As shown in Table 2, the chi-square test was used to compare the rehabilitation motivation of patients with stroke in different potential subgroups. The results showed statistically significant differences in age, monthly household income, medical expense payment method, ADL, degree of neurological deficit, and psychological distress (*P* < 0.05). Patients with stroke in the “Class 1” were mainly aged ≥60 years. Compared with Classes 2 and 3, they were older (*P* < 0.05) and had fewer ADL (*P* < 0.001), more severe neurological deficits (*P* < 0.05), and a larger proportion of patients with psychological distress (*P* < 0.001). Compared with Class 1, patients with stroke in the “Class 2” and “Class 3” had higher monthly household income (*P* < 0.05) and more ADL (*P* < 0.001).

**Table 2:** Differences among potential profiles of stroke patients rehabilitation motivations (*N* = 328).

**Variables**	**Classification**	**Profile 1 (*n* = 99)**	**Profile 2 (*n* = 101)**	**Profile 3 (*n* = 128)**	***X^2^***	***P***
Age	<60	30 (30.3)	41 (40.6)	62 (48.4)	7.615	**0.007**
	≥60	69 (69.7)	60 (59.4)	66 (51.6)		
Gender	Male	63 (63.6)	72 (71.3)	76 (64.3)	3.521	0.445
	Female	36 (36.4)	29 (28.7)	52 (35.7)		
Education level	Middle below	65 (65.7)	36 (35.6)	86 (57.0)	27.243	0.590
	High above	34 (34.3)	65 (64.4)	42 (43.0)		
Marital status	Unmarried	26 (26.3)	19 (18.8)	39 (30.5)	4.058	0.402
	Married	73 (73.7)	82 (81.2)	89 (69.5)		
Monthly income	<3,000	55 (55.6)	9 (8.9)	46 (35.9)	49.344	**0.009**
	≥3,000	44 (44.4)	92 (91.1)	82 (64.1)		
Medical insurance	Yes	91 (91.9)	98 (97.0)	125 (97.7)	5.098	**0.047**
	No	8 (8.1)	3 (3.0)	3 (2.3)		
Type of stroke	Ischemic	59 (59.6)	98 (97.0)	82 (64.1)	43.662	0.822
	Hemorrhage	40 (40.4)	3 (3.0)	46 (35.9)		
Time of stroke	≤6 months	45 (45.5)	79 (78.2)	62 (64.1)	27.708	0.946
	>6 months	54 (54.5)	22 (21.8)	66 (48.4)		
Number of strokes	One	42 (42.4)	81 (80.2)	57 (44.5)	37.884	0.947
	More than twice	57 (57.6)	20 (19.8)	71 (55.5)		
MBI	≥40	33 (33.3)	92 (91.1)	101 (78.9)	87.636	**<0.001**
	<40	66 (66.7)	9 (8.9)	27 (21.1)		
NIHSS	<5	16 (16.2)	70 (69.3)	48 (37.5)	59.415	**0.007**
	≥5	83 (83.8)	31 (30.7)	80 (62.5)		
Psychological distress	No	8 (8.1)	78 (77.2)	48 (37.5)	99.904	**<0.001**
	Yes	91 (91.9)	23 (22.3)	80 (62.5)		

The bold values indicate statistically significant results (*p* < 0.05).

### 3.3 Predictors of latent profiles of rehabilitation motivation of patients with stroke

Multivariate logistic regression analysis was used, with the latent profile of rehabilitation motivation as the dependent variable. “Class 1,” “Class 2,” and “Class 3” were assigned values of 1–3, respectively. Assignment of the independent variables is presented in Table 3.

**Table 3:** Variable assignment table.

**Variables**	**Assignment**
Age	1 = < 60, 2 = ≥60
Gender	1 = Male, 2 = Female
Education level	1 = Junior high school and below, 2 = Junior high school above
Marital status	1 = Unmarried/divorced/widowed, 2 = Married
Monthly income	1 = < 3,000, 2 = ≥3,000
Medical insurance	1 = Yes, 2 = No
Type of stroke	1 = Ischemic, 2 = Hemorrhage
Time of stroke	1 = ≤ 6 months, 2 = >6 months
Number of strokes	1 = One, 2 = More than twice
MBI(scores)	1 ≥ 40, 2 < 40
NIHSS (scores)	1 < 5, 2 = ≥5
Psychological distress	1 = No, 2 = Yes

**Figure 3:**
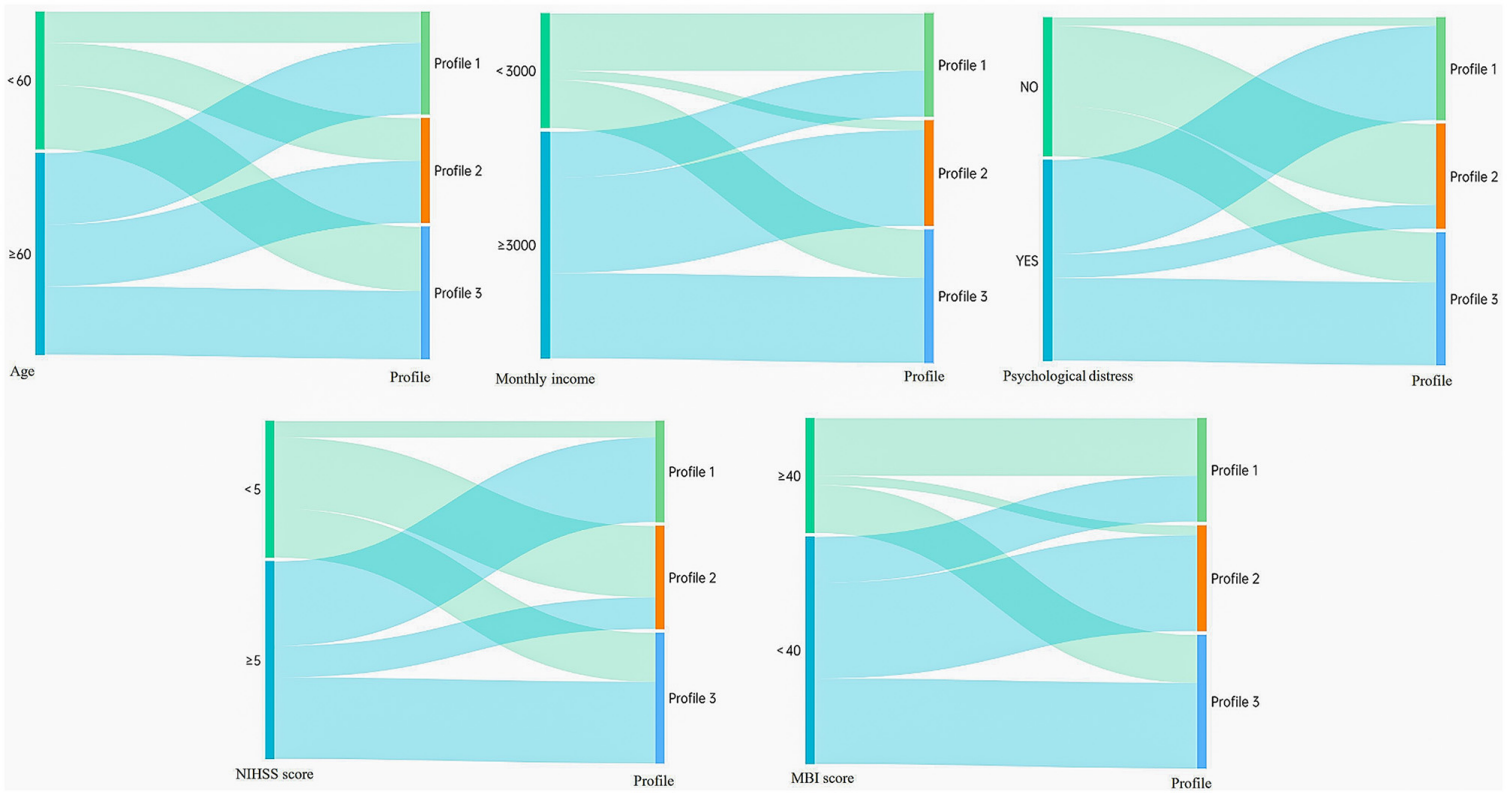
Sankey diagrams display the distribution of profiles based on their distinct characteristics (The left—hand nodes represent input feature classifications (salient feature variables), while the right—hand nodes represent output result classifications (three potential profiles). Each line signifies a data flow, with the line width indicating the magnitude of the data flow—the wider the line, the larger the numerical value. The direction of the lines reflects the flow direction of the data. *N* = 328).

As shown in Table 4, the results of the logistic regression analysis showed that age, monthly household income, ADL, degree of neurological impairment, and presence of psychological distress were influencing factors of the rehabilitation motivation level of patients with stroke (*P* < 0.05). Among these, monthly household income and ADL were protective factors for the rehabilitation motivation of patients with stroke, whereas age, degree of neurological impairment, and psychological distress were risk factors. Through visual analysis (Figure 3), the influences of five factors—age, monthly household income, ADL, degree of neurological impairment, and presence of psychological distress—on different latent subgroups were more clearly presented.

**Table 4:** Multiple logistic regression analysis of latent profiles of stroke patients' rehabilitation motivation (*N* = 328).

**Class**	**Variables**	**Estimate**	**Std. error**	***P***	**OR**	**95% CI for OR**
2	Monthly income < 5,000	−2.715	0.500	< 0.001	0.066	(0.025, 0.176)
	Monthly income ≥ 5,000					
	MBI ≥ 40	1.908	0.540	< 0.001	6.739	(2.339, 19.418)
	MBI < 40					
	NIHSS < 5	0.997	0.489	0.041	2.709	(1.040, 7.058)
	NIHSS ≥ 5					
	Psychological distress = NO	2.675	0.490	< 0.001	14.506	(5.547, 37.932)
	Psychological distress = YES					
3	Age < 60	0.931	0.337	0.006	2.537	(1.310, 4.911)
	Age ≥ 60					
	Monthly income < 5,000	−0.971	0.337	0.006	2.537	(1.316, 4.911)
	Monthly income ≥ 5,000					
	MBI ≥ 40	1.923	0.376	< 0.001	6.842	(3.277, 14.282)
	MBI < 40					
	Psychological distress = NO	1.331	0.453	0.003	3.786	(1.557, 9.205)
	Psychological distress = YES					

Class 1 was used as the reference.

1: Low rehabilitation motivation—intrinsic drive deficiency.

2: Moderate rehabilitation motivation—extrinsic drive stability.

3: High rehabilitation motivation—intrinsic drive sufficiency.

## 4 Discussion

### 4.1 The latent profiles of rehabilitation motivation for patients with stroke

LPA demonstrated significant advantages in exploring the heterogeneity of rehabilitation motivation among patients with stroke. Compared with traditional cluster analysis methods, LPA can precisely identify latent classes with distinct characteristics within the patient population, overcoming the limitations of traditional single-dimension assessments of rehabilitation motivation. Through a probabilistic modeling approach, LPA identified three motivational subgroups with unique psycho-behavioral characteristics: Low Rehabilitation Motivation-Intrinsic Drive Deficiency (Class 1, 30.2%), Moderate Rehabilitation Motivation-Extrinsic Drive Stability (Class 2, 39.0%), and High Rehabilitation Motivation-Intrinsic Drive Sufficiency (Class 3, 30.8%). These findings confirm the heterogeneous characteristics of rehabilitation motivation in patients with stroke and indicate that the rehabilitation motivation of most patients with stroke needs to be improved further, consistent with previous research results (Abdoust et al., 2024).

Class 1 are mainly elderly, with the proportion of patients aged 60 years and above reaching 69.7%, which is significantly higher than that in Classes 2 and 3, fully demonstrating the unique challenges faced by the elderly during rehabilitation (Tan et al., 2023). Furthermore, most patients in this class come from families with poor economic conditions, with 55.6% having a monthly household income below 3,000 yuan. This financial pressure may hinder the effective progress of their rehabilitation. Compared with Class 2 and Class 3, Class 1 are at a significant disadvantage in terms of ADL and neurological impairment, with statistical test results showing significant differences (*P* < 0.001, *P* < 0.05). This may be because these patients are affected by both family conditions and physiological functions, leading to low enthusiasm for autonomous participation in rehabilitation and a lack of goal-oriented behavior, which may directly affect the effectiveness of their active participation in rehabilitation exercises. Notably, in Class 1, the proportion of patients with psychological distress reached 91.9%, far exceeding that in Classes 2 (22.3%) and 3 (62.5%). This indicates that psychological factors may be key to maintaining moderate-to-high levels of rehabilitation motivation. High levels of psychological distress not only arise from the physical and mental stress caused by the disease itself but are also closely related to various factors, such as the patient's economic status, aging, and expectations of rehabilitation outcomes, consistent with the findings of Cheong et al. (2021). Therefore, there is an urgent need to focus on strengthening interventions that activate intrinsic motivation for this group. In contrast, Class 2 and Class 3 generally exhibited a more positive rehabilitation status. These two classes have a relatively higher proportion of young patients, and most come from families with better economic conditions, providing them with a more favorable rehabilitation environment and resources. Regarding ADL, neurological impairment, and psychological status, Class 2 and Class 3 were significantly better than Class 1. This further highlights the importance of intrinsic motivation and external support in the rehabilitation process of patients with stroke (Zielinska et al., 2015).

The findings of this study not only reveal the multidimensional characteristics of rehabilitation motivation but also provide valuable reference information for clinical practice. On the one hand, they assist medical professionals in identifying patient subgroups with significant differences in biological, psychological, and sociological characteristics, focusing on patient groups with moderate-to-low levels of rehabilitation motivation and their plasticity characteristics. On the other hand, they enhance medical professionals' sensitivity to patients' psychological states, enabling them to gain a deeper understanding of their rehabilitation motivation. When formulating personalized rehabilitation plans, medical professionals can fully consider the individual differences among patients, thereby effectively improving the rehabilitation outcomes of patients with stroke.

### 4.2 Factors predicting rehabilitation motivation of patients with stroke

#### 4.2.1 Predictive effects of biological factors on different latent classes of rehabilitation motivation in patients with stroke

The logistic regression analysis in this study revealed that, among the biological factors, ADL emerged as protective factors for rehabilitation motivation in patients with stroke (OR = 6.739, 6.842). Age and the degree of neurological impairment emerged as a risk factor for rehabilitation motivation in patients with stroke (OR = 2.537; OR = 2.709). Compared with Classes 2 and 3, elderly patients with stroke had a higher probability of being classified as Class 1, which confirmed the findings of previous studies that rehabilitation motivation levels are lower in elderly patients (Kim and Kim, 2017). In contrast, younger patients with stroke were more likely to exhibit stronger rehabilitation initiative. This may be because younger patients, who are the main source of family income and active participants in social activities, have a higher urgent need to restore their physical and mental functions as soon as possible, thus increasing their motivation to participate in rehabilitation exercises. However, elderly patients may be marginalized by the existing rehabilitation system owing to the dual impact of physiological decline and illness. Moreover, some elderly patients perceive their condition as a natural consequence of aging, and their daily life goals are limited to meeting basic survival needs, resulting in a lower rate of active rehabilitation (Morris et al., 2017). The results of our study further revealed that, compared with Class 1, patients with stroke with limited ADL (MBI < 40) and moderate-to-severe neurological impairment (NIHSS ≥ 5) were more likely to be categorized as Class 2 or Class 3, consistent with the findings of Dohnke et al. (2005) and Scorrano et al. (2018). This may be because neurological deficits directly impair the physical control and language communication abilities of patients with stroke, and these physiological limitations force them to have a stronger desire for rehabilitation. According to self-efficacy theory, when patients observe gradual improvements in their mobility during rehabilitation (such as improved MBI scores), their confidence in completing daily living tasks is significantly enhanced. This positive cognitive feedback creates a virtuous cycle that not only improves patients' compliance with rehabilitation treatment plans but also stimulates their intrinsic motivation to actively participate in rehabilitation exercises. In clinical practice, medical professionals should adopt stratified intervention strategies; for elderly patients, personalized rehabilitation programs should be developed, psychological support services should be strengthened, and assistance should be provided to help them rebuild a sense of meaning in life. For patients with an MBI < 40, priority should be given to rebuilding basic living skills, and their independent living abilities should be improved by using assistive devices and home environment modifications. For patients with NIHSS ≥ 5, neuromuscular re-education training should be intensified, and new technologies such as virtual reality should be introduced to enhance the fun of rehabilitation and improve patients' enthusiasm, thereby improving the effectiveness of rehabilitation training. In the future, medical professionals should adopt a dynamic assessment mechanism that adjusts intervention priorities based on patients' functional status to achieve precise rehabilitation services. Although demographic and disease-related factors, such as age, ADL, and degree of neurological impairment, are not easily modifiable, they can help medical professionals identify patients with different characteristics and manage them in a targeted manner.

#### 4.2.2 Predictive effects of psychological factors on different latent classes of rehabilitation motivation in patients with stroke

Logistic regression analysis in this study revealed that psychological distress emerged as a risk factor for rehabilitation motivation in patients with stroke (OR = 14.506, 3.786). Compared with Class 1, patients with stroke without psychological distress were more likely to be categorized as Class 2 or 3. This finding is consistent with those reported by Lee and Won (2022) and Tian et al. (2024). One possible reason is that patients with stroke without psychological distress may be better at expressing themselves and have stronger stress management abilities, often having greater motivation and confidence to cope with the disease, thus exhibiting higher rehabilitation motivation. The mechanism of action can be explained by the sub-theory of self-determination theory and Organismic Integration Theory (OIT): when basic psychological needs are hindered by emotional distress, patients have difficulty forming an intrinsic recognition of the value of rehabilitation and cannot achieve positive rehabilitation intentions (Ryan and Deci, 2017). Clinical data suggest that long-term negative emotions are more likely to be closely related to reduced rehabilitation activities and decreased motivation in patients and may even increase the risk of recurrence and death (Mutai et al., 2017). Psychological distress is prevalent in patients with stroke and mainly manifests as anxiety, depression, apathy, fear, frustration, and poor interpersonal relationships. It can occur during both the acute and recovery phases after a stroke, seriously affecting the rehabilitation process and quality of life of patients (Huang et al., 2017). Therefore, medical professionals should help patients overcome the negative emotions caused by the disease, provide systematic training in coping skills, and integrate psychological interventions as a phased or preparatory strategy with rehabilitation activities so that patients can maintain an optimistic mindset and positive attitude when participating in rehabilitation. In the future, psychological assessments should be incorporated into the routine rehabilitation process for patients with stroke, enabling dynamic monitoring of psychological status and immediate adjustment of intervention strategies, thus providing systematic support for improving rehabilitation motivation in patients with stroke.

#### 4.2.3 Predictive effects of sociological factors on different latent classes of rehabilitation motivation in patients with stroke

Logistic regression analysis in this study revealed that, among the sociodemographic factors, monthly household income emerged as a significant protective factor for rehabilitation motivation in patients with stroke (OR = 0.006, 2.537). Compared with Class 1, patients with higher monthly household income were more likely to be categorized as Class 2 or 3. This finding is consistent with the results reported by Seo and Yang (2020). A possible reason for this is that high-income families can provide better rehabilitation resources and economic security, effectively alleviating patients' concerns about poverty caused by illness. Such external support strengthens patients' extrinsic motivation. Moreover, this group may have more scientific access to medical information, which aligns with the core viewpoint of self-determination theory: when individuals acquire professional knowledge through interest-driven learning and practice autonomously, it triggers the transformation of cognitive drive into internal motivation, resulting in more positive behavioral initiative (Ryan and Deci, 2017). Clinical observations have also shown that patients from families with better economic conditions have a higher degree of disease awareness, better treatment compliance, and positive rehabilitation initiative (Cheong et al., 2021). Therefore, a stratified assessment system should be established for clinical interventions. For high-income patients, a “goal-oriented-achievement feedback” mechanism should be established, focusing on cultivating their intrinsic motivation for autonomous rehabilitation. For low-income patients, it is necessary to build a dual-track support system of “economic security and psychological empowerment” through various measures, such as preferential medical insurance policies and charitable assistance funds. This approach can alleviate economic pressure while stimulating treatment confidence, achieving the synergistic effects of internal and external motivations.

### 4.3 Limitations

This study has several limitations. First, the rehabilitation motivation data were solely collected from stroke patients in three hospitals, which restricts the generalizability of the findings. In the future, participants should be recruited from multiple centers, and independent samples should be used to validate the LPA (Latent Profile Analysis) results of this study. Second, cross-sectional studies are unable to ascertain the causal relationships between variables. Moreover, it is worth further exploring whether different rehabilitation motivation categories will improve or deteriorate over time. In the future, it is necessary to collect longitudinal data and use the Growth Mixture Model (GMM) to observe the evolution of rehabilitation motivation over time. This will facilitate better prediction of rehabilitation trajectories and the formulation of intervention strategies. Additionally, qualitative interviews should be combined to supplement the quantitative research results, providing deeper insights into patients' psychological experiences. Finally, the use of self-report questionnaires to collect data may be influenced by self -report bias. However, at the initial stage of the study design, to avoid further confounding effects of cognitive dysfunction on the assessment of self-report scales such as rehabilitation motivation, we excluded patients with cognitive dysfunction during the recruitment process. This is also a key limitation of our study design. In the future, consideration should be given to incorporating physiological indicators such as cognitive function and supplementing the research with electrophysiological and brain imaging techniques to further refine the study design and provide a more objective assessment of rehabilitation motivation in stroke patients.

## 5 Conclusion

Patients with stroke exhibit significant heterogeneity in rehabilitation motivation. Three distinct latent classes of rehabilitation motivation were identified: Class 1 (30.2%), Low Rehabilitation Motivation-Intrinsic Drive Deficiency; Class 2 (39%), Moderate Rehabilitation Motivation-Extrinsic Drive Stability; Class 3 (30.8%), High Rehabilitation Motivation-Intrinsic Drive Sufficiency. Age, monthly household income, ADL capacity, severity of neurological impairment, and psychological distress were significant predictors of latent class membership in stroke rehabilitation motivation. Healthcare providers should implement stratified intervention strategies tailored to specific motivational profiles to optimize clinical outcomes. This evidence-based, differentiated care approach ensures the effective mobilization of subjective initiative of patients with stroke in rehabilitation participation, thereby improving therapeutic adherence and functional recovery.

## Data Availability

The original contributions presented in the study are included in the article/supplementary material, further inquiries can be directed to the corresponding author.
